# Irritable Bowel Syndrome and Psychiatric Disorders in Pakistan: A Case Control Study

**DOI:** 10.1155/2012/291452

**Published:** 2012-02-28

**Authors:** Amna Subhan Butt, Mohammad Salih, Wasim Jafri, Javed Yakoob, Mohammad Wasay, Saeed Hamid

**Affiliations:** ^1^Section of Gastroenterology, Department of Medicine, The Aga Khan University & Hospital, Karachi 3500, Pakistan; ^2^Section of Neurology, Department of Medicine, The Aga Khan University, Karachi 3500, Pakistan

## Abstract

*Background*. The psychiatric disorders like anxiety and depression could have a profound influence on onset, expression, and course of Irritable bowel syndrome (IBS). *Aim*. To estimate the frequency and strength of association of common mental disorders (CMDs) in patients with IBS and patients with other chronic diseases, that is, migraine and hypertension. *Method*. This was a case control study. Individuals aged 18–70 years diagnosed as IBS were enrolled as cases. The control groups consisted of patients without IBS but diagnosed to have a chronic disease, that is, migraine or HTN. Self-Reporting Questonnaire-20(SRQ-20) was used as a screening tool for the detection of CMD. *Results*. 82 patients were enrolled in each group. Mean SRQ score was significantly higher in IBS group than controls (9.9 ± 4.5 versus 4.9 ± 3.6, *P* < 0.001). CMDs were more frequent (67.1% versus 22%) and the odds of CMD were 7.24 times higher among IBS patients than controls (95% CI 3.6–14.5, *P* < 0.001). No difference was found in frequency of CMDs among various subtypes of IBS. *Conclusion*. We found that CMDs are more common and strongly associated with IBS as compared to other chronic diseases. Early screening for CMDs might be useful for an effective management of IBS.

## 1. Introduction

Irritable bowel syndrome (IBS) is a common health problem which not only affects 10–20% of adult population worldwide [1] but also account for up to 25% outpatient workload of a gastroenterologist [2–4]. IBS is considered as a functional bowel disorder characterized by abdominal pain/discomfort which is relieved by defecation and associated with altered stool frequency and/or consistency, in the absence of structural, microbiological, and biochemical abnormalities [3, 5, 6]. The overall point prevalence of IBS in Asia varies from 4.4% to 10.4% in various communities [3, 7]. The exact prevalence of IBS in Pakistani population is unknown. However, the prevalence of IBS was found 34% in a study conducted in 508 college going students of Karachi, Pakistan [7]. Likewise, 45% of 1048 adults living in Karachi and Bahawalpur were found to be affected by IBS [8].

Despite conceptualization of several mechanisms, the exact cause of IBS remains uncertain. Currently, postulated mechanisms include exaggerated visceral hypersensitivity, gastrointestinal motor disturbances, and postinfectious IBS [5]. In addition, the brain gut axis has been suggested. Studies from different parts of the world have revealed underlying psychiatric disorders in 40–60% of IBS cases which is significantly higher than healthy control or controls with other chronic diseases [5, 9–15]. Moreover, in a study conducted in our hospital significantly high proportion of patients with functional dyspepsia were found to have common mental disorders as compared to controls (71.33% versus 15.33%, *P* < 0.001) [16]. Hence, the proper screening and management of associated common mental disorders in addition to the treatment is needed.

Common mental disorders (CMD) described as anxiety, depression, and somatoform disorders are the most frequent and disabling group of psychiatric states [17]. These CMDs like depression not only affect the quality of life but may potentially increase short-term mortality in cardiac patients [18]. Significant depressive symptoms have been reported in patients affected by chronic illness [19] for example, 18% of patients with migraine was found affected by CMD [20] and almost 50% patients with depression was found to have hypertension (HTN) [21]. In Pakistan, the overall reported prevalence of anxiety and depressive disorders is 34%, which is yet another challenge for a resource poor country like Pakistan [22].

Individuals with IBS incur more direct health care costs than non-IBS patients, particularly for their nongastrointestinal complaints including psychiatric symptoms [9, 23]. Frequency of symptoms experienced, concern of serious underlying illness, lower quality of life, and coexisting health problems lead to frequent visits and extended investigations of IBS patients [2]. Furthermore, coexisting CMD with IBS may further deteriorate health resulting in absenteeism at work, increased health care cost thus has significant implications on economics for a poor country like Pakistan. To date there is no published data regarding frequency of CMD in patients with IBS from Pakistan. Hence, identification of associated psychiatric disorders may help in treatment and counseling of patients with IBS through early psychological or psychopharmacological interventions [6]. Therefore, it is imperative to know the frequency and strength of association of CMD in patients with IBS from our region.

Thus, the current study is designed to estimate the frequency and strength of association of common mental disorders (CMDs) in Pakistani patients with irritable bowel syndrome (IBS) and patients with other chronic diseases, that are, migraine and hypertension. It is hypothesized that among IBS cases the CMD is greater than controls.

## 2. Methods

### 2.1. Study Design and Setting

Two parallel case control studies [16] were conducted in 2009 in Gastroenterology clinics of The Aga Khan University Hospital (AKUH), Karachi, Pakistan. First was the current study designed to evaluate the frequency of CMDs in patients with IBS as compared to controls. The AKUH is a tertiary care hospital where patients are referred from all over the country for treatment. The current study was conducted in outpatient Gastroenterology and Neurology clinics from January–September 2009. Individuals aged 18–70 years who could read and understand Urdu (national language) or English were offered to participate. Those who agreed to participate and met the eligibility criteria were enrolled with their consent.

### 2.2. Selection of Cases and Controls

Individuals visiting our Gastroenterology clinics and already diagnosed to have IBS by a qualified gastroenterologist at AKUH were recruited randomly as “cases”. However, those cases of IBS with diagnosed psychiatric disorders including CMD, other concomitant chronic diseases including HTN, DM, ischemic heart disease, and migraine, or had limited functional activity (i.e., functional class ≥2) were excluded. Diagnosis of IBS was made according to Room III criteria for IBS. Rome III criteria have been found useful and cost effective for diagnosing IBS in clinical practice, epidemiological surveys, physiological research and therapeutic trials [1, 6, 13]. Moreover, the IBS cases were also categorized into subtypes, that are, (1) IBS with constipation (IBS-C), (2) IBS with diarrhea (IBS-D), and (3) Mixed IBS (IBS-M) [1].

The control group was randomly selected from Neurology and Medicine clinics of AKUH. The control group comprised of patients who either have migraine diagnosed by a qualified neurologist or have HTN diagnosed based upon the Seventh Report of Joint National Committee (JNC VII) on Prevention, Detection, Evaluation, and Treatment of high blood pressure [24]. According to JNC VII Hypertension was defined as a mean of two or more properly measured seated BP readings of systolic BP ≥ 140–159 mmHg or diastolic BP ≥ 90–99 mmHg on each of two or more office visits [24]. Migraine was defined as lateralized, throbbing, or dull episodic headache occurring without preceding aura, sometimes associated with anorexia, nausea, vomiting, cognitive impairment, and blurring of vision [25].

Common mental disorders (CMD) describe the state of anxiety, depression, and psychosomatic disorders [17]. Based upon DSM IV criteria, diagnosis of major depression was made if an individual experienced five or more of the following symptoms for at least two consecutive weeks; (1) depressed mood, (2) loss of interest or pleasure in most or all activities, (3) insomnia or hypersomnia, (4) change in appetite or weight, (5) psychomotor retardation or agitation, (6) low energy, (7) poor concentration, (8) thoughts of worthlessness or guilt, and (9) recurrent thoughts about death or suicide. However, the individual diagnosed to have minor depression if experiences any two or four of the nine symptoms of major depression present most of the day, nearly every day, for at least two weeks, at least one being depressed mood or loss of interest/pleasure [26]. Anxiety disorder defined as an uncontrollable disposition to worry about one's welfare and that of one's immediate kin associated with wide range of physical and affective symptoms, changes in behavior, and cognition like arousal, vigilance, tension, irritability, and unrestful sleep [26, 27].

### 2.3. Screening for Common Mental Disorders

We used self-reporting questionnaire-20 (SRQ-20) which is one of the most widely used self-administered psychiatric questionnaires developed by Harding et al. [28] for World Health organization (WHO) to screen for CMD, especially in developing countries. It is available in at least 21 language translations including a validated Urdu version [29–32]. It has not only been validated but also adapted for cross-cultural use in Pakistan [30]. It is simple, easy to interpret and takes only few minutes to administer the questionnaire [30, 31, 33–36]. It consists of 20 questions and inquires about the presence of symptoms over the period of last 30 days [36]. Each item is scored 0 or 1. A score of 1 indicates the presence and 0 indicates the absence of symptoms with a maximum score of 20. Different cut off for SRQ scores has been used in different studies. However, a cutoff point of 8/9 (8 “yes” for a “control” and 9 “yes” for a “case”) is common with sensitivity of about 80% and specificity of 75% [30, 31].

Thus, eligible patients were interviewed by a single interviewer to screen them for CMDs by using SRQ-20. Moreover, information regarding age, gender, marital status for cases and controls, and type of IBS for cases were also collected.

This was a prospective, case control study, conducted by maintain compliance with the Helsinki Declaration. This was an interview based study and study participants were interviewed after their informed consent and there was no intervention involved.

### 2.4. Statistical Analysis

Sample size was calculated by using EPI Info version 6 [37]. Assuming the prevalence of CMDs 40% in IBS group [11] and 18% in the control group [19, 20] we require 148 (74 individuals in each group) to detect a difference in the proportion of CMDs in IBS group versus control group with 80% power, at 5% significance level. Adding 10% refusals to the calculated sample size, we required target sample size of 164 (82 subjects in each arm).

Data was analyzed in SPSS version 16.0. Mean ± standard deviation were calculated for quantitative variables (age, SRQ score) and proportions (percentage) were calculated for categorical data (gender, subtypes of IBS). The baseline characteristics were compared in the two groups by using independent student *t*-test for continuous variable and chi-square or Fisher exact test for categorical variables wherever appropriate. The association of age with CMDs was checked by stratifying cases and controls on age. A *P* value < 0.05 was considered statistically significant. All *P* values were single sided. Furthermore, binary logistic was performed to see association between CMDs and subtypes of IBS.

## 3. Results

A total of 164 patients; 82 patients in each group were enrolled. Out of 82 patients in control group, 42 patients had HTN and 40 had migraine. In IBS group, the proportion of IBS-constipation, IBS-diarrhea, and IBS-mixed were 37 (45.1%), 29 (35.4%), and 16 (19.5%), respectively. No difference was observed in mean age among patients with IBS and controls (43.1 ± 12.1 versus 39.9 ± 11.2 years, 95% CI 0.9–1.0, *P* = 0.08). There was male predominance in both groups. Moreover, CMDs were more frequent among males affected by IBS than controls (74.4% versus 58.5%, 95% CI 0.3–0.9, *P* = 0.03) (Table 1).

**Figure 1:**
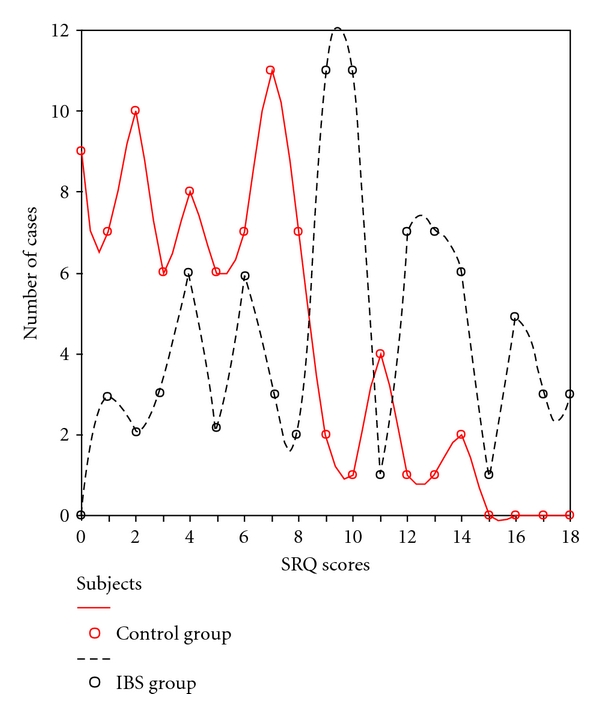
Comparison of SRQ-scores in IBS patients and controls.

Overall mean SRQ score was 7.4 ± 4.7 (range 0–18). Mean SRQ score was significantly higher in IBS group as compared to the control group (9.9 ± 4.5 versus 4.9 ± 3.6, 95% CI 1.22–1.44, *P* < 0.001) (Figure 1). Using the cut off 8/9 for SRQ score, common mental disorders were found in 55 (67.1%) of patients with IBS as compared to 18 (22%) of the control group. Moreover, the odds to be affected by CMDs were 7.2 times greater among IBS cases as compared to the control group (95% CI 3.6–14.5, *P* < 0.001). Furthermore, no difference was observed in frequency of CMDs among IBS cases and controls when both study groups were stratified on age (*P* value 0.6).

Binary logistic was also performed to see an association of CMD with subtypes of IBS. No difference was found in distribution of age and gender among different subtypes of IBS. Mean SRQ score was 10.62 ± 4.56, 8.52 ± 4.52, and 10.13 ± 4.12 in IBS-C, IBS-D, and IBS-mixed group, respectively (*P* = 0.16). Likewise, no difference was observed in frequency of CMD among patients with IBS-C, IBS-D, and IBS-mixed (27 (73.0%) versus 16 (55.2%) versus 12 (75.0%), *P* = 0.23) (Table 2).

## 4. Discussion

In this case control study, the distribution of age was comparable in both IBS and with chronic disease. Males were predominant among both cases and controls; however, the proportion of females was significantly higher in control group as compared to IBS group. Mean SRQ score and frequency of CMD were significantly higher in IBS group as compared to controls. We did not find difference in proportion of CMD and mean SRQ score among patients with IBS-C, IBS-D, and IBS-mixed. However, these findings were based upon post-hoc analysis and could be due to insufficient small sample size to assess this association.

The implications of our study are that there was a male predominance in both groups and CMDs were more frequent among male cases than controls. These findings are consistent with local studies conducted by Jafri and colleagues where IBS was found predominantly in males that is, 56% [8] and 48% (versus 42% in females) [7], respectively. Moreover, Healthcare seekers among IBS patients were predominantly males than females (56% versus 44%) [8]. Likewise, studies from neighboring country India have reported higher rates of IBS and dyspepsia among males than females [38, 39]. This might be due to possibility of male patients consulting doctors more often than females due to various sociocultural reasons here. Hence, in our study finding IBS more in males may suggest that the male preponderance for IBS is not a chance finding.

Our findings are consistent with Trikas and colleagues [10], who found depressive disorders more common among patients with IBS as compared to controls affected by cholelithiasis (36.1% versus 7.1%, *P* = 0.016). Likewise, 40 patients with IBS were compared with 32 patients with inflammatory bowel disease (IBD) and 34 healthy controls [40]. Prevalence of anxiety, depressive, and mood disorders as well as higher scores on anxiety and depression scales were found in IBS group as compared to the controls. Moreover, these findings were also true for patients with IBD as compared to healthy controls. In our study, the risk of CMD among patients with IBS was found 7.2 times higher than the controls. Moreover, we have used SRQ-20 to screen CMD in our study which is not only sensitive to detect depressive disorders but also detects anxiety and other somatoform disorders as well [19, 20]. However, conflicting results have also been reported in some studies.

In a cohort of 1037 young individuals in Dunedin, New Zealand [9], relationship between psychiatric disorders and IBS was investigated. Rome II and Manning criteria for IBS and modified version of Diagnostic interview Schedule was used to detect psychiatric disorders. No association was found between IBS and psychiatric disorders in this cohort. In contrast to this study where only young individuals of age 26 years were evaluated, individuals with age 18–70 years were evaluated in our study which may be a possible explanation for difference in frequencies of CMD in IBS. Another explanation may be that the first occurrence of IBS mostly occurs between 30–50 years of age [13]. Hence, the population under study represents a broad sample.

The limitations of our study include screening for CMD was based upon SRQ-20. It consists of symptoms experienced during last four weeks that could have led to some degree of recall bias. We found it better not match our cases and controls for age and gender. As age and gender are known risk factors for IBS and CMDs [5]. Moreover, prior local and regional studies had reported male preponderance for IBS. In case of matching for age and gender, association of age and gender could not be studied.

It has been suggested that cross-cultural research regarding molecular biology, genetic and psychosocial factors, disease manifestations, diagnosis and treatment, health seeking behaviors, health-related quality of life, all aspects of IBS could be affected by culture, and ethnicity and race. Hence, well-designed cross-cultural studies can help us to understand the epidemiology, pathophysiological mechanisms, and therapeutic interventions related to IBS [41, 42]. Pakistan is a developing country where political and economic instability is a major concern. Data from population-based studies indicate that a one-third of Pakistan's population has anxiety and depression [19, 39]. Despite the high burden of psychological problems, mental health is not given a priority in the National Health Policy and only 0.4% of health care budget is being allocated to mental health [43]. Although cost-effective treatments exist for these disorders, required trained mental-health professionals are not available to deal with them. Public funded primary health care is ineffective and underresourced.

## 5. Conclusion

We found that CMDs is more common and strongly associated with IBS as compared to other chronic diseases including migraine and HTN. Hence, early screening for CMDs in IBS patients might be useful for an effective management of IBS.

## Figures and Tables

**Table 1:** Comparison among patients with IBS and controls.

	Cases mean ± SD or *n* (%)	Controls mean ± SD or *n* (%)	95% confidence interval	*P* value
Age (years)	43.1 ± 12.1	39.9 ± 11.2	0.95–1.00	0.08

Gender				
Male	61 (74.4)	48 (58.5)	0.25–0.94	0.03
Females	21 (25.6)	34 (41.5)		

SRQ score	9.9 ± 4.5	4.9 ± 3.6	1.22–1.44	<0.001

CMDs				
Present	55 (67.1)	18 (22.0)	3.6–14.5	<0.001
Absent	27 (32.9)	64 (78.0)		

**Table 2:** Baseline demographic characteristics and CMD status of patients with different subtypes of IBS.

	IBS-C	IBS-D	IBS-mixed	*P* value
Gender*				
Male	25 (67.56%)	21 (72.4%)	15 (93.8%)	0.12
Female	12 (32.4%)	8 (27.6%)	1 (6.3%)	

Age (years)^†^	38.3 ± 10.13	42.6 ± 13.05	38.7 ± 9.67	0.26

Age categories (years)*				
18–34	14 (37.8%)	8 (27.6%)	6 (37.5%)	0.36
35–40	8 (21.6%)	9 (31.0%)	5 (31.3%)	
41–50	12 (32.4%)	5 (17.2%)	4 (25.0%)	
51–67	3 (8.1%)	7 (24.1%)	1 (6.3%)	

SRQ score^†^	10.62 ± 4.56	8.52 ± 4.52	10.13 ± 4.12	0.16

CMD present*	27 (73.0%)	16 (55.2%)	12 (75.0%)	0.23

*n (%), ^†^mean ± SD.

## Abbreviations

IBS: Irritable bowel syndrome
CMD: Common mental disorders
HTN: Hypertension
AKUH: Aga Khan University Hospital
IBS-C: IBS with constipation
IBS-D: IBS with diarrhea
IBS-M: Mixed IBS
JNC VII: Seventh Report of Joint National Committee VII
SRQ-20: Self-reporting questionnaire-20
WHO: World Health Organization.
